# Rumen Fluid Transplantation from *Allium mongolicum* Regel-Fed Donors Enhances Lamb Meat Quality and Reduces 4-Alkyl Branched-Chain Fatty Acids

**DOI:** 10.3390/foods15040701

**Published:** 2026-02-13

**Authors:** Xiaoyuan Wang, Guoli Han, Khas Erdene, Chen Bai, Qina Cao, Yankai Zheng, Terigele Li, Lahan Hai, Yande Fan, Yuqi Zhao, Xinyi Liu, Changjin Ao

**Affiliations:** 1 College of Animal Science, Inner Mongolia Agricultural University, Zhaowuda Road 306, Saihan District, Hohhot 010018, China; 2 Inner Mongolia Agriculture, Animal Husbandry Fishery and Biology Experiment Research Centre, Inner Mongolia Agricultural University, Zhaowuda Road 306, Saihan District, Hohhot 010010, China

**Keywords:** plant, mutton taint, carcass, *Longissimus thoracis* muscle, sheep

## Abstract

The extent of consumer approval for lamb is intimately connected to meat quality standards. Within this context, the distinctive ‘mutton taint’ serves as a critical benchmark for assessment, a characteristic that is largely governed by the concentrations of three fundamental branched-chain fatty acids (KBCFA), specifically 4-methyloctanoic acid (MOA), 4-ethyloctanoic acid (EOA), and 4-methylnonanoic acid (MNA). While *Allium mongolicum* Regel (AMR)—an Allium species prevalent in arid Asian regions known for its abundant bioactive constituents—is known to improve meat quality and mitigate these off-flavors, the potential mediating role of the rumen fluid in this process remains unclear. This study investigated whether rumen fluid transplantation (RFT) from AMR-fed donors could mimic the impacts of directly adding AMR to the diet on KBCFA accumulation and meat attributes. Thirty male lambs (23 ± 2 kg BW) were allocated at random into three distinct treatments (*n* = 10): a control set (CON), a dietary supplementation group administered 15 g/d of AMR (AMG), along with a rumen fluid transplantation treatment (RTG) inoculated with rumen fluid from AMR-fed donors. The carcass traits, physicochemical properties, and makeup of amino acids, as well as the fatty acid constitution of the *longissimus thoracis* muscle, were subjected to analysis. Data revealed that the levels of KBCFAs associated with off-flavors were markedly lowered in both the AMG and RTG. Specifically, decreases ranging from 49% to 64% were observed in MOA, EOA, and MNA concentrations (*p* < 0.05). Relative to the control group, drip loss and cooking loss were reduced in the treatment groups (*p* < 0.05), whereas ash (*p* = 0.047) and crude protein (*p* = 0.001) were increased. Moreover, the interventions improved the composition of essential amino acids (EAA), flavor-enhancing amino acids, and polyunsaturated fatty acids (PUFAs). In conclusion, rumen fluid transplantation effectively replicates the beneficial effects of dietary AMR on meat quality, particularly in reducing taint-related KBCFA. Such outcomes imply that rumen microbial communities likely act as a crucial mediator in controlling meat flavor.

## 1. Introduction

Three major 4-alkyl branched-chain fatty acids (KBCFA)—4-methyloctanoic acid (MOA), 4-ethyloctanoic acid (EOA), and 4-methylnonanoic acid (MNA)—serve as the primary contributors to the characteristic mutton taint [1]. Although some consumer segments appreciate this flavor, its intensity often lowers acceptance in mainstream markets, thereby constraining the commercial viability of lamb products [2]. To address these challenges, phytogenic feed additives have been proposed as a potential solution. These additives contain bioactive compounds capable of enhancing meat quality, improving nutritional profiles, and potentially mitigating mutton taint attributes [3,4].

*Allium mongolicum* Regel (AMR), an *Allium* species prevalent in the arid desert regions of Asia and Central Asia [5], contains abundant bioactive constituents, including flavonoids, polysaccharides, and organic acids [6]. Ding et al. [7] reported that AMR markedly decreased drip loss and cooking loss in lamb muscle. Additionally, supplementation with the ethanol extract of AMR decreased the content of C18:0 and the proportion of n-6 to n-3 fatty acids within the ovine *longissimus thoracis* (LT) tissues and increased the levels of C16:1 as well as C22:6 n-3 [8]. Moreover, AMR can modulate flavor formation in lamb by decreasing the precursors of key flavor compounds. For example, AMR lowered the concentrations of the abundance of MOA and MNA within the rumen and hepatic tissues of Small-tailed Han sheep, leading to reduced KBCFA deposition in the LT muscle; it also modulated the microbiota [8,9]. Another study confirmed that supplementation with AMR powder, water-soluble extract, or ethanol extract reduced KBCFA concentrations in the LT muscle [10]. Notably, some active compounds abundant in AMR can resist microbial degradation, improving their bioavailability and metabolic efficiency in the rumen [11]. This may inhibit microbial populations or enzymes that produce odorous precursors [12], indicating that remodeling the microbial community has the potential to influence lamb meat quality [13]. Nevertheless, earlier investigations have predominantly concentrated on the aggregate outcomes of AMR dietary inclusion. It remains ambiguous whether the decline in taint-associated KBCFAs is attributed directly to the uptake of AMR bioactive constituents or if it is indirectly facilitated by the modulation of rumen microbial populations.

Rumen fluid transplantation (RFT) has been applied to manage metabolic disorders in ruminants [14,15], this indirect approach to microbiota modulation is an effective nutritional intervention. It can alter rumen fermentation patterns [16], thus modulating the metabolic pathways of proteins and lipids [17], and ultimately improve fatty acid balance, flavor, antioxidant capacity, and overall meat characteristics [18]. While the benefits of AMR are well-documented, to our knowledge, no study has utilized RFT to decouple the microbial effects from the direct impacts of AMR supplementation. Therefore, it is necessary to verify whether the AMR-shaped microbiota alone is sufficient to reduce KBCFA deposition and improve meat quality in recipient lambs.

Therefore, we hypothesized that dynamic changes in rumen fluid are key factors influencing the content of KBCFA and quality of lamb meat. Consequently, the primary goal of this investigation was to partially transplant rumen fluid from donor lambs supplemented with AMR into recipient lambs and to compare this group with lambs directly fed AMR.

## 2. Materials and Methods

### 2.1. Ethics Statement for Animal Use

All animal-related procedures were reviewed and received official authorization from the Animal Welfare Committee at Inner Mongolia Agricultural University (Approval No. NND2022049) and were performed in strict accordance with the ethical directives issued by the Ministry of Science and Technology of China regarding the ethical treatment of animals in research.

### 2.2. AMR Powder Preparation

*Allium mongolicum* Regel was harvested from semi-arid steppe rangelands in Inner Mongolia, Alxa League, China, at the optimal harvest stage. The fresh specimens underwent dehydration in a forced-air oven (DHG-9070, Shanghai Yiheng Instrument Group Co., Ltd, Shanghai, China) set at 65 °C in order to maintain the integrity and bioactivity of the key constituents. The dried material was then ground using a DFT-300 mill (Shanghai Xinnuo Instrument Group Co., Ltd, Shanghai, China) and subsequently filtered through an 80-mesh screen (Shaoxing Shangyu Instrument Co., Ltd., Zhejiang, Shaoxing, China) to obtain AMR powder. The resulting powder was sealed in bags and kept refrigerated at 4 °C prior to utilization.

### 2.3. Experimental Design and Animals Management

This experiment took place at the Fuchuan Commercial Meat Breeding Sheep Co., a commercial farm located in Inner Mongolia, Bayannuur, China. The trial followed a two-phase design: Phase I established the rumen fluid donor animals, and Phase II involved rumen fluid infusion into the recipients. The overall experimental calendar spanned 135 days.

During Phase I, twelve three-month-old male crossbred lambs (Small-tailed Han × Dorper; BW 25 ± 1 kg) were chosen to act as the donors for rumen fluid. These animals received a standard basal ration enriched with 15 g daily of AMR powder over a 135-day period. Prior research indicates that supplementation with 15 g/day of AMR, under specific management conditions, alters the rumen microbiota and regulates KBCFA levels in LT muscle [19]. During Phase II, 30 male crossbred lambs (3 months; Small-tailed Han × Dorper; average BW 23 ± 2 kg) were allocated randomly to three experimental groups employing a fully randomized layout (10 animals per group): (1) CON, basal diet with oral saline infusion; (2) AMG group, fed a basal diet with the addition of 15 g/d AMR per lamb plus an oral saline infusion; (3) RTG group, fed a basal diet and receiving oral infusions of rumen fluid from the donor animals. Due to site constraints, animals in Phases I and II were managed under stall-feeding conditions that were similar but not identical. Lambs within each treatment were housed in three pens (containing 3, 3, and 4 lambs per pen, respectively). The individual animal was considered the experimental unit. Phase II consisted of an initial 15-day acclimatization phase succeeded by a feeding trial lasting 60 days, totalling 75 days. The acclimatization stage for Phase II began on the 60th day of Phase I; AMR feeding and RFT were initiated on day 16 of Phase II.

**Figure 1:**
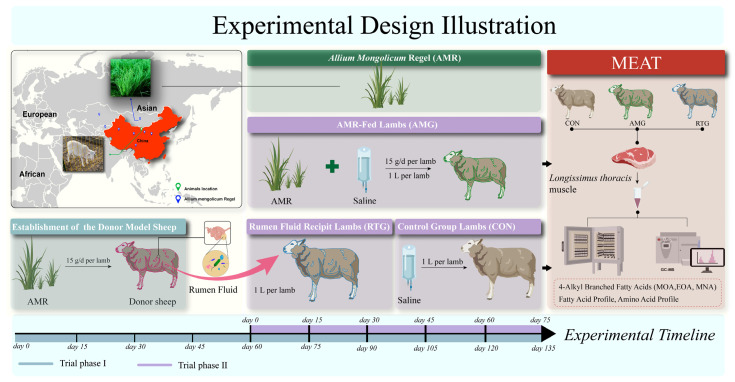
Experimental design illustration. The schematic layout illustrates the experimental arrangement from left to right, indicating animal placement and the administration of the *Allium mongolicum* Regel (AMR) supplement. To create the rumen fluid donor model, lambs were administered a daily dosage of 15 g AMR (Pink). Subjects allocated to the AMG treatment were provided the standard ration fortified with 15 g/day of AMR alongside an oral administration of 1 L saline solution; those consuming the basal diet with 15 g/d AMR supplementation and receiving 1 L of oral saline were designated as the AMG cohort (Green); the RTG cohort consisted of lambs maintained on the basic diet which were administered an oral gavage of 1 L rumen liquor harvested from the AMR-fed donors (Blue). The adaptation phase for the second stage of the trial commenced promptly on the 60th day of Phase I. Subsequent to this acclimatization period, transfers of rumen fluid were executed every 15 days, amounting to five distinct procedures.

Rumen fluid transplantation was used as an intervention and was performed according to the procedure described by Liu et al. [16]. Collection of ruminal fluid was carried out from all donor lambs (Phase I) 2 h before morning feeding. At the time of collection, donors were fasted to ensure a stable rumen environment and reduce the risk of reflux. Rumen contents were collected via a ruminal cannula (A1164K, Wuhan Anscitech Farming Technology Co., Ltd, Hubei, Wuhan, China) equipped with a metal filter to separate the liquid phase from large feed particles. From each donor, the initial 100 mL fraction was disposed of to mitigate potential salivary contamination. The subsequent 300 mL aliquot of ruminal fluid was collected from each donor. The fluids from all 12 donors were immediately combined into a sizable, CO_2_-flushed vessel and mixed thoroughly to create a composite inoculum. On transplantation days, recipient lambs (Phase II) were fasted for ten hours prior to infusion. The composite rumen fluid was infused immediately after preparation. Subjects belonging to the CON and AMG cohorts received an administration of 250 mL saline solution, whereas lambs in RTG received 250 mL of the pooled rumen fluid. The transplantation procedures were carried out at 15-day intervals for four rounds. Throughout Phase II, the CON and AMG lambs were given a total aggregate volume amounting to 1 L of saline, and RTG lambs received 1 L rumen fluid. The nutrient composition and fatty acids profiles of the basal diet are listed in Table 1 and Table 2, respectively, while the methodological details are illustrated in Figure 1.

### 2.4. Sample Collection

At the end of the feeding trial, the animals underwent a 12 h fasting period (water provided ad libitum) prior to slaughter. In each group, the heaviest and lightest animals were excluded to minimize body-weight variation. From the remaining animals, six lambs per group were randomly selected for sampling. Animals were transported to a commercial meat processing facility (Inner Mongolia Little Sheep Meat Industry Co., Ltd., Inner Mongolia, Bayannur, China) where they were slaughtered according to standard humane procedures and local regulations.

Hot carcass weight was recorded immediately after slaughter, and samples of the LT muscle were dissected from the left carcass flank at the 12th–13th rib interface at 45 min postmortem. For meat quality measurements, LT samples intended for pH and color analyses were kept at 4 °C and evaluated at 45 min and 24 h postmortem. For the physicochemical traits, fatty acid profile, and KBCFA quantification, roughly 150 g of LT tissue was cleaned of observable connective tissues and subcutaneous fat layers, then vacuum-sealed, flash-frozen using liquid nitrogen, and vacuum packaged at −80 °C pending analysis.

### 2.5. Chemical Analysis of Feed Samples

Representative feed samples were collected from each diet throughout the experimental period. Specifically, weekly samples were taken, pooled by every 7 days for each treatment, and stored at −20 °C until analysis. Before analysis, samples were oven-dried at 65 °C for 48 h until they achieved a stable weight, followed by grinding to fit through a 1.0 mm screen using a laboratory mill.

Nutrient composition was determined following AOAC (2005) [20] methods: dry matter (DM; AOAC method 930.15), ash content (method 942.05), crude protein (CP; calculated as Kjeldahl N × 6.25; method 990.03), as well as ether extract (EE; method 920.39). Calcium (Ca; method 968.05) and phosphorus (P; method 975.16) were quantified utilizing a microplate reader (Multiskan FC, Thermo Fisher Scientific Inc., Waltham, MA, USA). The content of neutral detergent fiber (NDF; method 2002.04) and acid detergent fiber (ADF; method 973.19) were determined via an Ankom fiber analysis system (ANKOM A200i, ANKOM Technology, Macedon, NY, USA).

### 2.6. Assessment of Growth Parameters

The determination of growth indices followed the protocol described previously [21]. Briefly, the lambs’ body mass was measured before the morning meal at the study’s onset (initial body weight, IBW) and conclusion (final body weight, FBW), and subsequently every two weeks to track growth trends. The average daily gain (ADG) was computed based on the equation below:

(1) ADG (kg/d) = FBW-IBW/65 (d)

The amounts of feed offered and the remaining leftovers were logged daily per pen. The daily dry matter intake (DMI) was determined via the subsequent equation:

(2) DMI (kg/d) = feed offered−feed refusals/hand of sheep

The feed-to-gain ratio (F/G) was calculated as follows:

(3) F/G = DMI/ADG

### 2.7. Carcass Attributes and LT Muscle Characteristics

Immediately following slaughter, the dressed carcass weight was documented in adherence to the protocol of Coleman et al. [22]. After chilling, backfat thickness (BFT) was measured by vertical probing positioned at the interface of the 12th and 13th ribs utilizing a calibrated vernier caliper (Mitutoyo 500–196, Mitutoyo Precision Measuring Instruments, Kanagawa, Kawasaki, Japan; accuracy ± 0.02 mm). For eye muscle area (EMA), carcasses were transversely sectioned at the 12th rib; the exposed muscle surface was traced onto tracing paper, and dimensions were measured in triplicate. EMA was then calculated using the following formula:

(4) $$\mathrm{EMA}\;({\mathrm{cm}}^{2})\;=\;0.7\;\times \;H\;\times \;W$$

where H is the maximum vertical height, and W represents the maximum horizontal width.

### 2.8. pH and Meat Color Measurements in the LT Muscle

A handheld pH-STAR device (2128W, Spectrum, California, USA) was utilized to measure LT muscle pH in triplicate at 45 min and 24 h post-slaughter. The device was calibrated at 20 ± 1 °C with standard buffer solutions (pH 4.01 and 7.00). After being refrigerated for 1 h at 4 °C, the color (DP18198, Beijing Yodp Technology Co., Ltd, Beijing, China) indices (L^*^, a^*^ and b^*^) of the LT muscle were evaluated per the method of Pestana et al. [23]. Measurements were taken at three distinct surface points per sample using a calibrated colorimeter (DS-700C-1, CHNSpec Technology Co., Ltd., Zhejiang, Hangzhou, China), and the mean of the three readings was used for analysis. The device settings included a D65 illuminant, 2° observer, an 11 mm aperture size, an 8 mm measuring zone, and a pulsed xenon lamp. Furthermore, saturation (Chroma, C^*^) and hue angle (*h*^°^) were computed using the equations below:

(5) $$C^{*}\;=(a^{*2}\;+b^{*2}{)}^{0.5}$$

(6) $$h\text{°}=\arctan(\frac{b^{*}}{a^{*}})\;\times \;\frac{180}{\Pi }$$

### 2.9. Evaluation of Physical Quality Traits in LT Muscle Samples

The assessment of drip loss, cooking loss, and shear force was conducted in accordance with the protocols [24,25]. Each LT muscle sample (2 × 2 × 4 cm) was recorded for initial weight (W1) and subsequently hung inside airtight vessels at 4 °C for a 24 h duration, ensuring no contact with the container walls. Upon completion, surface fluid was absorbed using filter paper, after which the concluding mass (W2) was documented. The drip loss percentage was derived using the equation below:

(7) $$\mathrm{Drip}\;\mathrm{loss}\;\left(\%\right)\;=\;\left(W1\text{–}W2\right)/W1\;\times \;100\%$$

Vacuum-sealed samples were submerged in a water bath preheated to 80 °C. Heating was maintained until the central core temperature reached 70 °C. After cooling and surface blotting, specimens were weighed again to obtain W4. Cooking loss percentage was determined by

(8) $$\mathrm{Cooking}\;\mathrm{loss}\;\left(\%\right)=\left(W3\text{–}W4\right)/W3\;\times \;100\%$$

To quantify shear force values, visible connective tissue and fat were removed from each LT muscle sample, followed by cooking. Measurements were conducted using a specialized meat quality testing instrument for meat quality analysis (C-LM4, Tenovo International Co., Limited, Beijing, China). The muscle was sectioned into square-shaped strips of 1 cm^2^ cross-sectional size, aligned parallel to the fibers, followed by shear application. The mean shear force (N) per sample was calculated as the average of five peak-force measurements.

### 2.10. Proximate Composition in the LT Muscle

For proximate composition, LT muscle samples were trimmed of fascia and adipose tissue and freeze-dried (FDL-1000, Tokyo EYELA Co., Ltd., Tokyo, Japan). CP (990.03) and EE (920.39) were determined according to AOAC (2005) [20]. The moisture level was quantified via desiccation via oven-drying at 105 °C until a constant mass was reached. Ash levels were assessed by combustion in a muffle furnace heated to 600 °C until weight stabilization.

### 2.11. Quantification of KBCFA Levels in the LT Muscle

The qualitative and quantitative analysis of KBCFA was conducted following the protocol of Watkins et al. [26] utilizing a GC-MS platform system (Trace 1310 GC coupled with ISQ LT Single-Quadrupole Mass Spectrometer, Thermo Fisher Scientific, Waltham, MA, USA). Chromatographic separation was accomplished using a DB-WAX capillary column (30 m × 0.25 mm × 0.25 μm; Agilent Technologies). Injection was carried out using a 1 μL aliquot in splitless mode, while the injection inlet was maintained at a constant temperature of 250 °C. High-purity helium (>99.999%) functioned as the carrier gas, supplied at a consistent flow of 1.0 mL/min. The oven temperature program was set to operate as follows: initiated at 50 °C (2 min hold), subsequently increasing to 180 °C at a rate of 10 °C/min, and ultimately ramping to 240 °C at 5 °C/min with a final holding of 10 min. Mass spectrometry analysis utilized electron ionization (EI) at 70 eV. The temperatures for the ion source and transfer line were set to 230 °C and 250 °C, respectively.

To optimize detection sensitivity for the analytes of interest, data collection was conducted employing Selected Ion Monitoring (SIM) mode. In this research, the specific ions *m*/*z* 99 and *m*/*z* 113 exhibited the greatest abundance and specificity. The limits of detection (LOD) were determined to be 0.15 mg/kg (MOA), and 0.11 mg/kg for both EOA and MNA. The limits of quantification (LOQ) were computed as threefold the LOD values.

### 2.12. Fatty Acid Profile in the LT Muscle

Total lipid isolation was performed on roughly 2.0 g of freeze-dried LT muscle tissue, strictly adhering to the chloroform–methanol (2:1, *v*/*v*) procedure described by Folch et al. [27]. Following solvent evaporation under nitrogen flow and weighing, the lipids underwent a dual-stage transmethylation process to generate methyl esters of fatty acids (Sinopharm Group Beijing Co., Ltd, Beijing, China; FAMEs). Specifically, approximately 20 mg of extracted lipid was dissolved into 2 mL of 0.5 M methanolic NaOH (Sinopharm Group Beijing Co., Ltd, Beijing, China) and heated at 80 °C for a duration of 15 min to complete saponification. Upon cooling, methylation was induced, adding 2 mL of 14% BF_3_-methanol (Sinopharm Group Beijing Co., Ltd, Beijing, China), followed by incubation at 80 °C for 5 min. The resulting FAMEs were then recovered using 2 mL of n-hexane. Internal standardization was achieved using Undecanoic acid (C11:0).

The analysis of the FAMEs was conducted on a gas chromatography platform (Trace 1310, Thermo Scientific^TM^, Waltham, MA, USA) equipped with an FID and employing a fused silica capillary column (SP-2560, 100 m × 0.25 mm × 0.20 μm; Supelco, Bellefonte, PA, USA). The injector and detector temperatures were fixed at 260 °C. The oven program initiated at 140 °C (5 min hold), increased to 240 °C at 4 °C/min, and ended with a 15 min hold. Nitrogen acted as the carrier gas, supplied at 1.0 mL/min with a 100:1 split ratio. The identification of specific fatty acids relied on matching their retention times against a commercial FAME reference standard (Supelco 37 Component FAME Mix, Sigma-Aldrich).

### 2.13. Amino Acid Composition of the LT Muscle

The freeze-dried LT muscle samples (0.50 g and 0.20 g) were used to determine the concentrations of amino acids from hydrolyzed protein (HPAA) and free amino acids (FAA), respectively. For protein-hydrolysate amino acids, a 0.50 g sample aliquot was purged with nitrogen for 2 min, followed by acid hydrolysis utilizing 6 M HCl (Sinopharm Group Beijing Co., Ltd, Beijing, China) at 110 °C for a 24 h period. Once cooled to ambient temperature, the mixture underwent filtration followed by dilution to a total volume of 25 mL in a volumetric flask. A 0.50 mL portion underwent evaporation under a mild nitrogen flow until nearly dry. To ensure complete acid removal, the addition of 200 μL ultrapure water and the evaporation process were performed 2–3 times. The resulting residue was reconstituted in 2.5 mL of 0.02 mol/L HCl with the aid of 5 min of sonication. Subsequently, a 1.0 mL aliquot was filtered through a 0.22 μm membrane prior to analysis.

For FAA, samples were homogenized in 0.02 M HCl followed by centrifugation at 13,000× *g* (15 min, 4 °C). The obtained supernatant was mixed with 0.50 mL of 10% (*w*/*v*) sulfosalicylic acid for deproteinization and subjected to centrifugation again under identical conditions. The clarified supernatant was subsequently passed through a 0.22 μm filter for analysis.

Quantification was achieved via ion-exchange chromatography coupled with ninhydrin detection utilizing an L-8900 amino acid analyzer (Hitachi, Japan), in the procedure detailed by Li et al. [28]. The categories analyzed included: essential amino acids (EAA: Thr, Val, Met, Ile, Leu, Phe, Lys, His, Arg) and umami-related amino acids (UAA: Asp, Glu, Gly, Ala, Arg, Met).

### 2.14. Statistical Analysis

Data regarding the meat quality traits, physicochemical indices, KBCFA, and fatty/amino acid profiles were analyzed using one-way ANOVA with the SAS software package (v. 9.21; SAS Institute Inc., Cary, NC, USA). Before conducting the analysis, the Shapiro–Wilk test was employed to confirm the assumptions of normal distribution and homogeneity of variance. Data were screened for outliers using externally studentized residuals. Regarding growth metrics, given the absence of significant variations in IBW across groups, a one-way ANOVA was conducted without including initial weight as a covariate. Duncan’s multiple range test was utilized to compare group means, chosen for its efficacy in distinguishing between multiple treatment conditions. The results are presented as mean values. A probability level of *p* < 0.05 was considered statistically significant. The statistical analysis followed the linear model structure described by Hu et al. [29], as detailed below:

(9) Yij=μ+Ti+εij

In this equation, *Yij* signifies the response variable, where *μ* denotes the overall mean (*n* = 6), *Ti* indicates the fixed effect corresponding to the specific diet treatment (CON, AMG, RTG), and *εij* represents the random error term.

## 3. Results

### 3.1. Growth Performance

Consistent with prior findings [21], the addition of AMG and RTG led to a significant enhancement in lamb growth performance (Table 3). Relative to the CON cohort, both supplemented groups demonstrated substantially higher FBW (*p* < 0.05) and ADG (*p* < 0.001), while no statistical disparity was found between the two treatment groups.

### 3.2. Carcass Traits and Meat Quality Attributes of the LT Muscle

Table 4 displays the data regarding the carcass characteristics of the LT muscle. Regarding the carcass traits, the EMA exhibited a significant elevation in the AMG and RTG treatment groups compared to the CON cohort (*p* = 0.034). Conversely, statistical analysis revealed no marked disparities in terms of dressed carcass weight or BFT across the different dietary treatments (*p* > 0.05). With respect to the meat quality parameters, a notable reduction in drip loss was observed for both the AMG and RTG treatments when compared with the CON group (*p* = 0.020). In contrast, other physical parameters, including the pH_45 min_ and pH_24 h_ values and parameters such as cooking loss and shear force remained statistically comparable and unaffected by the experimental diets (*p* > 0.05). Furthermore, there were no significant differences in the L^*^, a^*^, and b^*^ values across the groups (*p* > 0.05), whereas the CON group presented the most desirable hue angle (*h*^°^), succeeded by the AMG and RTG groups, respectively (*p* > 0.05).

### 3.3. Physicochemical Properties in the LT Muscle

Table 5 summarizes the physicochemical attributes of the LT muscle. Statistical analysis indicated that moisture and EE levels remained consistent and demonstrated no statistical differences among the dietary groups (*p* > 0.05). Conversely, the experimental diets exerted a significant impact on both ash (*p* = 0.047) as well as CP composition (*p* = 0.001). Specifically, the AMG and RTG cohorts exhibited elevated concentrations of ash and CP relative to the CON controls (*p* < 0.05); however, no statistical distinction was found when comparing the AMG and RTG treatment cohorts directly.

### 3.4. Level of KBCFA in the LT Muscle

The concentrations of MOA, EOA, and MNA in the LT muscle were markedly downregulated in both the AMG and RTG groups compared to the control (*p* < 0.001, Table 6). Both treatments demonstrated similar efficacy in mitigating these unpleasant flavor precursors, with no statistical disparity detected between them (*p* > 0.05).

### 3.5. Fatty Acid Composition of the LT Muscle

Table 7 illustrates the fatty acid makeup of the LT muscle. To be specific, the total saturated fatty acids (SFAs) decreased significantly in both the the AMG and RTG cohorts relative to the CON group, whereas total monounsaturated fatty acids (MUFAs) exhibited a notable rise (*p* < 0.01). With respect to specific fatty acids, both AMG and RTG interventions led to significant declines in various SFAs, such as C14:0 and C16:0, along with C17:0, C20:0, and C20:1 (*p* < 0.05). In contrast, desirable unsaturated fatty acids, especially C16:1 and C18:1 n-9 c, showed significantly elevated levels in the treatment groups (*p* < 0.01). Although the general outcomes were comparable, certain variations specific to the treatments were observed. The concentration of C18:2 n-6 c was significantly reduced solely in the AMG cohort (*p* = 0.037), remaining stable in the RTG cohort. Moreover, a direct comparison indicated that the AMG group possessed higher C17:1 values yet a lower C20:1 level versus the RTG group (*p* < 0.05).

### 3.6. Amino Acid Composition of the LT Muscle

Table 8 presents the hydrolyzed protein amino acid data for the LT muscle. Overall, the dietary interventions positively impacted the amino acid balance. To be specific, the concentrations of total (TAA), essential (EAA), and non-essential amino acids (UAA) were significantly increased in both the AMG and RTG groups compared to the CON group (*p* < 0.01). Nevertheless, no significant statistical differences were found between the two treatment groups themselves. With respect to specific amino acids, both treatments resulted in a significant elevation of Asp, Gly, Leu, Met, and Arg levels versus the CON cohort (*p* < 0.05). For Thr and Ile, the RTG group exhibited levels that significantly exceeded those of the CON group (*p* < 0.05), while the AMG group displayed mid-range values that showed no statistical distinction from either the control or RTG treatments. In contrast, the statistical analysis indicated no significant disparities among the groups regarding Ser, Glu, Ala, Cys, Val, Tyr, Phe, Lys, His, and Pro (*p* > 0.05).

Table 9 summarizes the free amino acid (FAA) profile of the LT muscle. A clear stepwise increase was noted in TAA, EAA, and UAA levels, with the highest values in the AMG group, followed by the RTG group, and the lowest in the CON group (*p* < 0.01). This pattern was also consistent for individual amino acids such as Ser, Glu, Gly, Ala, Val, Leu, Tyr, and Phe; specifically, the AMG group showed significantly higher levels than the RTG group, which in turn exceeded the CON group (*p* < 0.01). On the other hand, for Asp, Lys, and His, both treatment groups displayed significantly higher concentrations than the CON group (*p* < 0.01), but did not differ significantly from each other. Notably, the treatments elicited differential effects on Met and Ile. Specifically, a significant increase in Met concentration was found solely in the RTG group compared to the CON group (*p* < 0.01), whereas a significant elevation in Ile levels was specific to the AMG group (*p* < 0.01). Statistical testing revealed no significant differences in the Thr and Arg concentrations among the various dietary regimens (*p* > 0.05).

## 4. Discussion

**Figure 2:**
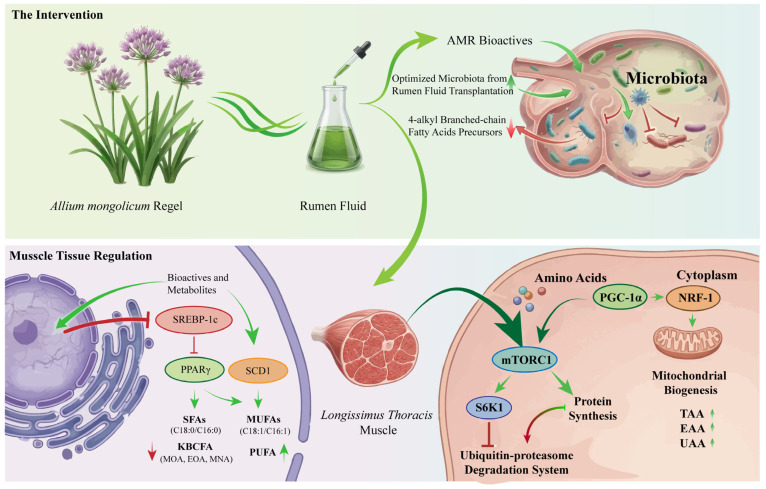
Potential mechanisms driving the impact of rumen fluid transplantation on meat attributes as well as 4-alkyl branched-chain fatty acids. This figure schematically illustrates the potential sequential impact of transferring rumen fluid from donor sheep receiving 15 g/day of *Allium mongolicum* Regel on the rumen microbial community, which may subsequently affect the synthetic and metabolic functions in muscle [30,31,32,33,34].

Compared to the control group, RFT treatment did not alter meat color or shear force. However, it significantly affected the fatty acid and amino acid compositions of the meat, particularly the KBCFA profiles associated with mutton taint. As depicted in the hypothetical mechanism (Figure 2), taking into account the regulation of the rumen ecosystem, transplantation appears to decrease the availability of key precursors for KBCFA. Although the precise molecular signaling pathways have not been fully elucidated in the current experimental design, it nevertheless provides critical insights into this ‘rumen–muscle’ axis. We further hypothesized that this microbial intervention would specifically enrich flavor-related volatile compounds, which the current results support and will be discussed in more detail below.

### 4.1. Growth Performance

Both AMR supplementation and RFT using rumen fluid from AMR-fed donor sheep significantly increased FBW and ADG. Similarly, Ding et al. [7] observed that dietary AMR supplementation improved ADG in lambs, aligning with our findings. Compounds in members of the *Allium* genus, including organosulfur compounds, flavonoids, saponins, and polysaccharides, can exert selective inhibitory and stimulatory effects on microbes, reducing the activity of methanogenic archaea and some protozoa [35] while enriching fiber-degrading and volatile fatty acid-producing taxa such as *Ruminococcus*, *Fibrobacter*, *Prevotella*, *Selenomonas*, and *Succinivibrionaceae* [36]. RFT directly inoculates recipient sheep with an established microbial consortium and, by shifting the microbiota and its metabolic profile, can rapidly reconfigure fermentation patterns [5,37], thereby producing effects similar to feeding AMR.

### 4.2. The Longissimus Thoracis Characteristics

Previous investigations in lamb nutrition have primarily focused on AMR and its processed derivatives, including ethanol extracts and flavonoid-enriched fractions [9,38]. Ding et al. [7] found comparable meat color parameters but significantly reduced drip loss in AMR-supplemented groups, consistent with our results. Redoy et al. [39] observed elevated EE content with Allium sativum supplementation in sheep, though without significant effects on cooking loss or drip loss. In contrast, Liu et al. [40] reported reduced cooking loss in AMR-fed Angus calves. The efficacy of AMR may be attributed to its bioactive components. Organosulfur compounds may inhibit proteolytic enzymes, preserving muscle proteins, and mitigate lipid peroxidation via free radical scavenging, thereby maintaining membrane integrity and water-holding capacity. Polyphenolic constituents such as O-glucuronide conjugates and 3-O-rutinosides exhibit antioxidative activity via free radical scavenging, lipid-peroxidation suppression, and enhancement of endogenous antioxidant enzyme activity [41,42]. Because oxidative stress acts as a primary factor contributing to muscle protein degradation [43], these actions may help increase CP retention in muscles. Furthermore, flavonoids can promote myofiber hypertrophy by inhibiting muscle protein degradation and enhancing mitochondrial biogenesis. For example, apigenin increases mitochondrial number and volume and upregulates mitochondrial biogenesis-related genes, including PGC-1α and NRF-1, thereby improving myofiber size and morphology [44]. Collectively, these findings provide initial evidence that AMR and RFT are effective interventions to improve meat quality traits. However, further controlled studies are needed to confirm causality, rule out carcass-weight confounding, and clarify the underlying mechanisms.

### 4.3. 4-Alkyl Branched-Chain Fatty Acid Concentration

As major biomarkers for lamb meat flavor characterization [45], KBCFA have been a major focus of our research program. In the present study, both AMR supplementation and RFT reduced KBCFA concentrations in the LT muscle, yielding a low-KBCFA phenotype within the AMG and RTG cohorts. These findings corroborate previous reports showing that AMR-derived extracts lower the amount of KBCFA in lamb tissues. Liu et al. [5] reported decreased MOA with AMR flavonoid supplementation, whereas Zhao et al. [9] demonstrated that an ethanol-based extract of AMR reduced hepatic MOA and MNA without affecting EOA. The observed reductions may relate to antioxidant mechanisms, as AMR flavonoids, anthocyanins, and other polyphenols possess strong free radical-scavenging activity [10]; the antioxidant efficacy of these compounds correlates with their hydroxyl group configuration [30], enabling interruption of lipid-peroxidation chain reactions [31]. The decrease in KBCFAs in RTG further indicates that rumen fluid transplantation can reshape microbial communities and expedite recovery from dysbiosis [16], potentially altering KBCFA metabolism through donor–recipient microbiome transfer [14]. These findings support AMR supplementation and RFT as effective strategies for modulating KBCFA in lambs. Nonetheless, mechanistic studies are needed to identify the specific microbes and metabolic pathways that govern KBCFA regulation.

### 4.4. Fatty Acid Characteristics

Fatty acid metabolism functions as a crucial factor defining both the quality and flavor attributes in ruminant products [46]. Epidemiological evidence indicates that higher intakes of certain SFAs, particularly C14:0 and C16:0, exhibit a positive correlation with the risk of developing cardiovascular diseases [47]. In contrast, species-typical mutton flavor is more closely linked to specific branched-chain fatty acids, notably MOA and EOA, and to lipid-oxidation products formed during cooking [46,48,49,50]. Within the current investigation, C16:0, C18:0, as well as C18:1 n-9 c, were the predominant fatty acids, consistent with recent lamb profiles [40]. Both AMG and RTG showed higher MUFA and lower SFA levels, with a significant increase in cis-9 C18:1. We attribute these shifts, at least in part, to AMR polyphenols with strong antioxidative activity, which may attenuate the biohydrogenating activity of bacteria such as *Butyrivibrio* and thereby favor greater deposition of unsaturated fatty acids [17]. In addition, flavonoids and polyphenols such as quercetin can modulate key transcriptional regulators of lipid metabolism, including PPARγ and SCD1, influencing fatty acid desaturation and oxidative pathways; SCD1 catalyzes the conversion transforming C18:0 and C16:0 into their corresponding forms C18:1 and C16:1 [32,51]. Meanwhile, active compounds such as isorhamnetin and anthocyanins can suppress SREBP-1c, a lipogenic transcription factor that normally upregulates SCD1 [32]. Acting in concert, these regulators may enhance apparent Δ9-desaturase efficiency and promote the coordinated formation of MUFA, including C18:1 n-9 c and C16:1 [52]. Concurrently, C16:0 decreased—a change generally viewed as beneficial for human health—whereas increases in oleic acid are often associated with improved eating quality. A significant reduction in the n-6/n-3 ratio was observed within the AMG cohort, a profile typically considered advantageous for health and potentially reflecting altered rumen biohydrogenation and enhanced preservation and deposition of n-3 PUFA [53,54].

### 4.5. Amino Acid Characteristics

The amino acid composition of lamb meat plays a vital role in determining its nutritional value as well as flavor potential [55]. FAA contribute directly to taste—Glu and Asp impart umami, while Gly and Ala add sweetness—and act as precursors for volatile compounds formed during Maillard and Strecker reactions in cooking, yielding aldehydes, ketones, sulfur- and nitrogen-containing heterocycles, and aromatic derivatives that shape distinctive mutton notes [56,57,58]. The sulfur-containing amino acids Met and Cys, along with yield methional and other sulfur heterocycles [59], enhance aroma complexity, whereas branched-chain amino acids (BCAAs), specifically Val, Leu, and Ile, generate Strecker aldehydes such as 3-methylbutanal and 2-methylbutanal, associated with roasted and nutty flavors [60,61]. Our findings indicate an enrichment of TAA, EAA, and UAA in the LT muscle with both interventions, across protein-bound and free pools, suggesting improved muscle protein accretion and greater availability of taste precursors. The levels of Glu, Asp, Gly, Ala, Lys, Phe, Leu, Ile, Val, and Met increased. AMG showed the highest concentrations, and the RTG group was intermediate between the AMG and CON groups. Over 40% of hydrolyzed amino acids mirrored patterns in FAA [8], suggesting that dietary or microbiome interventions can influence muscle protein deposition and the postmortem release of taste-active precursors. AMR provides flavonoids, anthocyanins, and organosulfur compounds that can selectively suppress hyper-deaminating bacteria and protozoa, reducing ruminal amino acid deamination and NH_3_-N accumulation, while enriching peptide- and amino acid-utilizing taxa such as *Prevotella* and *Succinivibrionaceae* [33].

Interestingly, unlike reports for other polyphenol-rich forages, our fermentation data indicated a reduction in the proportion of propionate (unpublished data), suggesting a shift toward rumen fermentation pathways favoring lipid-synthesis precursors (acetate and butyrate) while preserving nitrogen in microbial biomass [62,63]. Polyphenol–protein interactions may also transiently protect dietary protein from rapid ruminal degradation, thereby increasing rumen-undegraded protein flow and enhancing small-intestinal absorption of limiting amino acids such as Lys and Met [64]. Post-absorptively, bioactives with antioxidant and anti-inflammatory capabilities may mitigate muscle protein degradation proteins through the ubiquitin–proteasome and autophagy–lysosome pathways, while promoting mTORC1–S6K1-mediated protein synthesis, leading to net protein accretion [33,34]. The observed enrichment of Lys is nutritionally relevant, as greater Lys availability supports protein synthesis in adults and may help mitigate age-related muscle loss [64]. Remarkably, RTG replicated much of AMG’s amino acid signature without direct AMR intake, indicating successful transfer of a nutritionally optimized rumen microbiota that increased microbial protein output and reduced nitrogen loss, thereby enhancing amino acid supply to muscles. The lower free-amino acid levels in RTG compared with AMG likely reflect the absence of systemic host-level modulation by AMR.

### 4.6. Limitations

Nevertheless, certain limitations of the present study warrant acknowledgment. Our intervention effectively mediated changes in flavor precursors and meat quality, but it remains unclear whether the applied frequency of intervention represents the optimal strategy for establishing stable and long-term microbial colonization. The dynamic nature of microbial ecosystems suggests that a chosen frequency might induce transient shifts rather than permanent integration. Future investigations should therefore explore the kinetics of microbial colonization to ascertain the minimum effective frequency required to sustain the desired phenotypic traits. Collectively, these findings indicate that targeted modulation or transplantation of the rumen microbiota can co-optimize the nutritional and sensory amino acid landscape of lamb, offering a potential interventation for improving meat quality without continuous dietary supplementation.

## 5. Conclusions

To conclude, the transfer of rumen fluid from donor sheep supplemented with *Allium mongolicum* Regel successfully lowered the levels of 4-alkyl branched-chain fatty acids linked to mutton odor while simultaneously yielding moderate enhancements in meat quality attributes.

## Figures and Tables

**Table 1:** Composition and nutrient levels of the basal diet (%, dry matter basis).

Items (Basal Diet)	Content	Items (*Allium mongolicum* Regel)	Content
Ingredients		Chemical composition ^3^	
Corn stalk silage	19.67	Flavonoids	24.33
Alfalfa meal	16.35	Organic acids and their derivatives	20.02
Corn stalk	10.01	Nucleotides and their derivatives	12.87
Wheat bran	7.30	Amino acids	14.07
Corn	25.20	Hydroxycinnamoyl derivatives	5.22
Soybean meal	14.53	Amino acids derivatives	3.41
Extruded soybeans	1.83	Phenol amine	2.37
Premixes ^1^	2.48	Vitamins	1.40
NaCl	1.50	Choline	1.09
Limestone	1.13	Lipids	3.96
Total	100.00	Quinic acids and their derivatives	0.49
Nutrient levels ^2^		Others	10.77
Metabolic Energy (MJ, kg)	11.82		
Crude protein	14.50		
Ether extract	2.50		
Neutral detergent fiber	34.60		
Acid detergent fiber	16.43		
Organic matter	85.00		
Ca	2.00		
P	0.80		

^1^ The nutritional premix provided the following constituents per kilogram of the total ration: Mn, 30.00 mg (as manganese source); 25.00 mg Fe (as ferrous sulfate); 29.00 mg Zn (provided by zinc sulfate); 8.00 mg Cu (as copper sulfate); 0.10 mg Co (supplied as cobalt sulfate); and 0.45 mg I (as potassium iodide). The diet was fortified with vitamins including 3200 IU vitamin A, 1200 IU vitamin D, and 20 IU vitamin E. ^2^ Metabolizable energy (ME) values were determined following the calculation method of Ding et al. [7], whereas all remaining nutrient levels represent analyzed data. ^3^ The chemical compositions of AMR were identified through experimental measurement.

**Table 2:** Fatty acid composition of the basal diet (μg/g, dry matter basis).

Items (Basal Diet) ^1^	Content	Items (Basal Diet) ^1^	Content
C4:0	9.61	C18:2 n-6 c	5087.88
C6:0	103.73	C20:0	28.20
C8:0	30.86	C20:1	15.15
C10:0	32.46	C18:3 n-3	300.16
C11:0	- ^2^	C21:0	159.45
C12:0	244.76	C20:2	29.36
C14:0	110.80	C22:0	22.23
C14:1	147.64	C20:4	14.97
C15:0	5.89	C23:0	23.17
C16:0	2399.93	C22:2	6.61
C16:1	84.33	C24:0	29.78
C17:0	44.24	C24:1	5.20
C17:1	47.26	C22:6	36.68
C18:0	516.45	Σ SFAs	3498.72
C18:1 n-9 t	43.03	Σ MUFAs	2845.64
C18:1 n-9 c	2503.02	Σ PUFAs	5403.96

^1^ Σ SFAs: the sum of saturated fatty acids; Σ MUFAs: the sum of monounsaturated fatty acids; Σ PUFAs: the sum of polyunsaturated fatty acids. ^2^ “-” indicate not detected.

**Table 3:** Effects of supplementation of *Allium mongolicum* Regel or rumen fluid transplantation on the growth performance of lambs [21].

Items	Treatments ^1^			SEM	*p*-Value
	CON	AMG	RTG		
IBW (kg)	22.47	22.53	24.18	0.65	0.128
FBW (kg)	44.78 ^b^	48.33 ^a^	49.87 ^a^	0.69	0.005
DMI (kg/d)	1.61	1.56	1.58	0.05	0.702
ADG (kg/d)	0.34 ^b^	0.42 ^a^	0.45 ^a^	0.01	<0.001
F/G	4.91	4.66	5.41	0.17	0.163

^1^ CON: fed with a basal diet and received a total of 1 L saline; AMG: fed with a basal diet with 15 g/d per lamb of *Allium mongolicum* Regel powder and received a total of 1 L saline; RTG: fed with a basal diet and received a total of 1 L rumen fluid from donor sheep; IBW: initial body weight; FBW: final body weight; DMI: dry matter intake; ADG: average daily gain; F/G: feed-to-gain ratio. Values within the same row bearing distinct superscript letters denote statistically significant variations (*p* < 0.05).

**Table 4:** Effects of supplementation of *Allium mongolicum* Regel or rumen fluid transplantation on qualitative characteristics of the *longissimus thoracis* muscle of lambs.

Items	Treatments ^1^			SEM	*p*-Value
	CON	AMG	RTG		
pH_45 min_	6.55	6.26	6.48	0.09	0.091
pH_24 h_	5.37	5.38	5.40	0.04	0.748
L*	38.14	37.82	34.76	1.37	0.185
a*	18.31	17.62	19.54	0.94	0.359
b*	2.94	2.47	1.85	0.33	0.091
C*	18.60	17.83	19.63	0.92	0.396
*h*°	7.82	7.19	5.57	0.68	0.092
Drip loss (%)	4.24 ^a^	2.45 ^b^	1.91 ^b^	0.55	0.020
Cooking loss (%)	36.2	35.36	36.91	1.25	0.685
Shear force (N)	107.84	118.32	108.96	3.74	0.136
Dressed carcass weight (kg)	22.29	22.82	23.45	0.49	0.283
BFT (mm)	7.93	8.57	8.88	0.60	0.546
EMA (cm^2^)	18.93 ^b^	23.53 ^a^	22.61 ^a^	1.09	0.034

^1^ CON: fed with a basal diet and received a total of 1 L saline; AMG: fed with a basal diet with 15 g/d per lamb of *Allium mongolicum* Regel powder and received a total of 1 L saline; RTG: fed with a basal diet and received a total of 1 L rumen fluid from donor sheep; L*: lightness; a* = redness; b*: yellowness; C*: saturation; *h*°: hue angle; BFT: backfat thickness; EMA: eye muscle area. Values within the same row bearing distinct superscript letters denote statistically significant variations (*p* < 0.05).

**Table 5:** Effects of supplementation of *Allium mongolicum* Regel or rumen fluid transplantation on the physical–chemical parameters of the *longissimus thoracis* muscle of lambs.

Items	Treatments ^1^			SEM	*p*-Value
	CON	AMG	RTG		
Moisture (%)	75.72	74.28	76.77	3.11	0.853
Ash (%)	0.97 ^b^	1.21 ^a^	1.23 ^a^	0.07	0.047
EE (%)	3.59	3.39	3.92	0.55	0.807
CP (%)	19.15 ^b^	21.39 ^a^	23.18 ^a^	0.65	0.001

^1^ CON: fed with a basal diet and received a total of 1 L saline; AMG: fed with a basal diet with 15 g/d per lamb of *Allium mongolicum* Regel powder and received a total of 1 L saline; RTG: fed with a basal diet and received a total of 1 L rumen fluid from donor sheep; EE: ether extract; CP: crude protein. Values within the same row bearing distinct superscript letters denote statistically significant variations (*p* < 0.05).

**Table 6:** Effects of supplementation of *Allium mongolicum* Regel or rumen fluid transplantation on MOA, EOA, and MNA concentration of the *longissimus thoracis* muscle of lambs (mg/kg).

Items	Treatments ^1^			SEM	*p*-Value
	CON	AMG	RTG		
MOA	12.34 ^a^	4.38 ^b^	4.55 ^b^	0.71	<0.001
EOA	4.13 ^a^	1.87 ^b^	1.81 ^b^	0.20	<0.001
MNA	3.77 ^a^	1.91 ^b^	1.90 ^b^	0.16	<0.001

^1^ CON: fed with a basal diet and received a total of 1 L saline; AMG: fed with a basal diet with 15 g/d per lamb of *Allium mongolicum* Regel powder and received a total of 1 L saline; RTG: fed with a basal diet and received a total of 1 L rumen fluid from donor sheep; MOA: 4-methyloctanoic acid; EOA: 4-ethyloctanoic acid; MNA: 4-methylnonanoic acid. Values within the same row bearing distinct superscript letters denote statistically significant variations (*p* < 0.05).

**Table 7:** Effects of supplementation of *Allium mongolicum* Regel or rumen fluid transplantation on fatty acid composition of the *longissimus thoracis* muscle of lambs (mg/g, dry matter basis).

Items	Treatments ^1^			SEM	*p*-Value
	CON	AMG	RTG		
C10:0	0.56	0.69	0.64	0.05	0.275
C12:0	0.51	0.51	0.43	0.05	0.458
C14:0	11.25 ^a^	8.16 ^b^	8.16 ^b^	0.25	<0.001
C15:0	0.50	0.54	0.53	0.08	0.929
C16:0	95.12 ^a^	73.05 ^b^	70.91 ^b^	4.34	0.014
C17:0	4.55 ^a^	2.45 ^b^	2.38 ^b^	0.35	0.007
C18:0	36.29	33.93	33.52	2.38	0.690
C20:0	0.38 ^a^	0.12 ^b^	0.13 ^b^	0.03	0.002
C14:1	0.34	0.33	0.24	0.05	0.427
C16:1	3.63 ^b^	5.18 ^a^	5.01 ^a^	0.25	0.008
C17:1	1.23 ^ab^	1.48 ^a^	1.00 ^b^	0.18	0.046
C20:1	0.23 ^a^	0.14 ^c^	0.17 ^b^	0.01	0.001
C18:1 n-9 t	6.78	7.00	7.13	0.71	0.943
C18:1 n-9 c	60.44 ^b^	87.08 ^a^	83.26 ^a^	1.99	<0.001
C18:2 n-6 t	0.39	0.39	0.41	0.03	0.944
C18:2 n-6 c	19.21 ^a^	15.89 ^b^	17.35 ^ab^	0.68	0.037
C18:3 n-3	0.73	0.73	0.68	0.05	0.737
C20:3 n-6	0.35	0.37	0.37	0.01	0.650
C20:4	6.67	7.09	7.04	0.11	0.071
n-6/n-3	0.03 ^a^	0.02 ^b^	0.03 ^a^	0.001	0.044
Σ FAs	249.21	245.16	239.39	6.30	0.573
Σ SFAs	149.16 ^a^	119.45 ^b^	116.71 ^b^	4.79	0.005
Σ MUFAs	72.67 ^b^	101.21 ^a^	96.81 ^a^	2.07	<0.001
Σ PUFAs	27.36	24.47	25.85	0.73	0.081

^1^ CON: fed with a basal diet and received a total of 1 L saline; AMG: fed with a basal diet with 15 g/d per lamb of *Allium mongolicum* Regel powder and received a total of 1 L saline; RTG: fed with a basal diet and received a total of 1 L rumen fluid from donor sheep; Σ FAs: the sum of all fatty acids; Σ SFAs: the sum of saturated fatty acids; Σ MUFAs: the sum of monounsaturated fatty acids; Σ PUFAs: the sum of polyunsaturated fatty acids. Values within the same row bearing distinct superscript letters denote statistically significant variations (*p* < 0.05).

**Table 8:** Effects of *Allium mongolicum* Regel supplementation or rumen fluid transplantation on hydrolyzed protein amino acid composition in *longissimus thoracis* muscle of lambs (g/100 g, dry matter basis).

Items	Treatments ^1^			SEM	*p*-Value
	CON	AMG	RTG		
Asp	4.73 ^b^	5.88 ^a^	6.10 ^a^	0.18	<0.01
Thr	2.75 ^b^	3.09 ^ab^	3.32 ^a^	0.11	0.011
Ser	2.58	2.50	2.69	0.09	0.368
Glu	10.11	10.28	10.65	0.27	0.377
Gly	2.48 ^b^	2.82 ^a^	2.89 ^a^	0.10	0.015
Ala	3.92	3.69	3.91	0.13	0.417
Cys	0.61	0.60	0.52	0.08	0.656
Val	2.67	2.70	2.77	0.08	0.630
Met	1.21 ^b^	1.60 ^a^	1.60 ^a^	0.07	<0.01
Ile	2.34 ^b^	2.55 ^ab^	2.67 ^a^	0.08	0.031
Leu	4.79 ^b^	5.32 ^a^	5.30 ^a^	0.13	0.025
Tyr	2.17	2.29	2.30	0.06	0.260
Phe	2.79	2.94	3.05	0.09	0.156
Lys	4.96	5.13	5.18	0.17	0.633
His	2.50	2.42	2.61	0.07	0.168
Arg	3.51 ^b^	4.03 ^a^	4.17 ^a^	0.15	0.021
Pro	2.92	2.72	2.79	0.08	0.209
TAA	56.71 ^b^	60.70 ^a^	62.91 ^a^	0.75	<0.01
EAA	27.52 ^b^	29.78 ^a^	30.68 ^a^	0.38	<0.01
UAA	25.80 ^b^	28.16 ^a^	29.43 ^a^	0.52	<0.01

^1^ CON: fed with a basal diet and received a total of 1 L saline; AMG: fed with a basal diet with 15 g/d per lamb of *Allium mongolicum* Regel powder and received a total of 1 L saline; RTG: fed with a basal diet and received a total of 1 L rumen fluid from donor sheep; TAA: the total of all hydrolyzed protein amino acids; EAA: essential amino acid: Thr, Val, Met, Ile, Leu, Phe, Lys, His and Arg; UAA: umami amino acids, the sum of the umami-tasting amino acid: Asp, Glu, Gly, Ala, Arg, Met. Values within the same row bearing distinct superscript letters denote statistically significant variations (*p* < 0.05).

**Table 9:** Effects of *Allium mongolicum* Regel supplementation or rumen fluid transplantation on free amino acid composition in *longissimus thoracis* muscle of lambs (mg/100 g, dry matter basis).

Items	Treatments ^1^			SEM	*p*-Value
	CON	AMG	RTG		
Asp	2.72 ^b^	4.40 ^a^	4.86 ^a^	0.25	<0.01
Thr	23.18	23.77	20.92	1.24	0.30
Ser	13.41 ^c^	16.52 ^a^	14.94 ^b^	0.47	<0.01
Glu	61.72 ^c^	83.47 ^a^	71.01 ^b^	2.52	<0.01
Gly	37.27 ^c^	69.32 ^a^	51.99 ^b^	1.79	<0.01
Ala	95.98 ^c^	144.87 ^a^	127.23 ^b^	3.59	<0.01
Cys	-	-	-	-	-
Val	9.93 ^c^	14.07 ^a^	12.84 ^b^	0.38	<0.01
Met	4.49 ^b^	5.16 ^b^	6.04 ^a^	0.28	<0.01
Ile	6.00 ^b^	8.24 ^a^	6.21 ^b^	0.19	<0.01
Leu	10.86 ^c^	15.44 ^a^	13.41 ^b^	0.30	<0.01
Tyr	7.46 ^c^	10.55 ^a^	9.40 ^b^	0.34	<0.01
Phe	26.78 ^c^	41.88 ^a^	37.17 ^b^	0.95	<0.01
Lys	88.09 ^b^	128.51 ^a^	125.60 ^a^	3.66	<0.01
His	17.79 ^b^	26.07 ^a^	28.83 ^a^	1.49	<0.01
Arg	24.57	25.98	23.83	0.52	0.153
Pro	-	-	-	-	-
TAA	431.79 ^c^	620.86 ^a^	543.53 ^b^	8.29	<0.01
EAA	213.21 ^c^	291.72 ^a^	264.10 ^b^	5.89	<0.01
UAA	225.86 ^c^	335.59 ^a^	282.58 ^b^	5.82	<0.01

- indicates not deleted; ^1^ CON: fed with a basal diet and received a total of 1 L saline; AMG: fed with a basal diet with 15 g/d per lamb of *Allium mongolicum* Regel powder and received a total of 1 L saline; RTG: fed with a basal diet and received a total of 1 L rumen fluid from donor sheep; TAA: the total of all hydrolyzed protein amino acids; EAAs: essential amino acids (comprising Thr, Val, Met, Ile, Leu, Phe, Lys, His, and Arg). UAA refers to umami-related amino acids, calculated as the aggregate of Asp, Glu, Gly, Ala, Arg, and Met. Values within the same row bearing distinct superscript letters denote statistically significant variations (*p* < 0.05).

## Data Availability

The original contributions presented in the study are included in the article, further inquiries can be directed to the corresponding author.

## Abbreviations

The following abbreviations are used in this manuscript:

*a**	Redness
ADF	Acid detergent fiber
ADG	Average daily gain
AMR	*Allium mongolicum* Regel
*b**	Yellowness
BFT	Backfat thickness
Ca	Calcium
CP	Crude protein
DM	Dry matter
DMI	Dry matter intake
EAAs	Essential amino acids
EE	Ether extreat
EI	Electron ionization
EMA	Eye muscle area
EOA	4-ethyloctanoic acid
FAAs	Free amino acids
FAMEs	Fatty acid methyl esters
FID	Flame ionization detector
F/G	Feed-to-gain ratio
GC	Gas chromatography
GC-MS	Gas chromatography–mass spectrometry
HPAAs	Hydrolyzed protein amino acids
KBCFAs	Three key 4-alkyl branched-chain fatty acids
*L**	Lightness
LT	*Longissimus thoracis*
MNA	4-methylnonanoic acid
MOA	4-methyloctanoic acid
MUFAs	Monounsaturated fatty acids
NDF	Neutral detergent fiber
P	Phosphorus
PUFAs	Polyunsaturated fatty acids
RFT	Rumen fluid transplantation
SFAs	Saturated fatty acids
SIM	Selected ion monitoring
TAAs	Total amino acids
UAAs	Umami amino acids
